# Care-seeking and delay of care during COPD exacerbations

**DOI:** 10.1038/s41533-022-00269-9

**Published:** 2022-02-15

**Authors:** Emily R. Locke, Jessica P. Young, Catherine Battaglia, Tracy L. Simpson, Ranak Trivedi, Carol Simons, John C. Fortney, Paul Hebert, Erik R. Swenson, Jeffrey Edelman, Vincent S. Fan

**Affiliations:** 1 Center of Innovation for Veteran-Centered and Value-Driven Care VA Puget Sound Health Care System, Seattle, WA United States; 2 Eastern Colorado VA Health Care System, Aurora, CO United States; 3 University of Colorado Anschutz Medical Campus, Aurora, CO United States; 4 Center of Excellence in Substance Addiction Treatment and Education (CESATE), VA Puget Sound Health Care System, Seattle, WA United States; 5 Department of Psychiatry, University of Washington School of Medicine, Seattle, WA United States; 6 Center for Innovation to Implementation, VA Palo Alto Health Care System, Palo Alto, CA United States; 7 Department of Psychiatry and Behavioral Sciences, Stanford University, Stanford, CA United States; 8 Department of Health Systems and Population Health, University of Washington, Seattle, WA United States; 9 Pulmonary and Critical Care, VA Puget Sound Health Care System, Seattle, WA United States; 10 Department of Medicine, University of Washington, Seattle, WA United States

**Keywords:** Chronic obstructive pulmonary disease, Respiratory signs and symptoms, Outcomes research, Health services

## Abstract

Patients who receive earlier treatment for acute exacerbations of chronic obstructive pulmonary disease (COPD) have a better prognosis, including earlier symptom resolution and reduced risk of future emergency-department visits (ED) or hospitalizations. However, many patients delay seeking care or do not report worsening symptoms to their healthcare provider. In this study, we aimed to understand how patients perceived their breathing symptoms and identify factors that led to seeking or delaying care for an acute exacerbation of COPD. We conducted semistructured interviews with 60 individuals following a recent COPD exacerbation. Participants were identified from a larger study of outpatients with COPD by purposive sampling by exacerbation type: 15 untreated, 15 treated with prednisone and/or antibiotics in the outpatient setting, 16 treated in an urgent care or ED setting, and 14 hospitalized. Data were analyzed using inductive content analysis. Participants were primarily male (97%) with a mean age of 69.1 ± 6.9 years, mean FEV_1_ 1.42 (±0.63), and mean mMRC dyspnea of 2.7 (±1.1). We identified 4 primary themes: (i) access and attitudinal barriers contribute to reluctance to seek care, (ii) waiting is a typical response to new exacerbations, (iii) transitioning from waiting to care-seeking: the tipping point, and (iv) learning from and avoiding worse outcomes. Interventions to encourage earlier care-seeking for COPD exacerbations should consider individuals’ existing self-management approaches, address attitudinal barriers to seeking care, and consider health-system changes to increase access to non-emergent outpatient treatment for exacerbations.

**Clinical Trial Registration** NCT02725294

## Introduction

Persons with chronic obstructive pulmonary disease (COPD) frequently experience acute exacerbations that are associated with decreased quality of life^1^ and can lead to emergency-department (ED) visits and hospitalizations^2^. The economic impact of severe exacerbations is significant. In the United States in 2010 there were an estimated 1.5 million ED visits and 699,000 COPD hospitalizations with a mean cost of $8,228 per hospitalization^3,4^.

Patients with exacerbations may require pharmacologic treatment, including bronchodilators, corticosteroids or antibiotics^5^. Earlier treatment can lead to more rapid symptom recovery and fewer ED visits and hospitalizations^1,6^. However, in one study, more than 70% of patients presenting to the ED with a COPD exacerbation waited more than 24 h before seeking care, thereby increasing the risk of hospitalization^6^. Moreover, an estimated 40% of patients do not report all their exacerbations to their healthcare providers, also increasing the risk of COPD hospitalization^1^.

COPD self-management programs that include an action plan with prescriptions for home antibiotics and prednisone for treatment of exacerbations have been shown to improve quality of life and reduce hospitalizations^7,8^. In a randomized controlled trial of a COPD exercise program where all participants had a COPD action plan, those initiating prednisone and antibiotics within 3 days of new respiratory symptoms had more rapid resolution of symptoms, but no difference in healthcare utilization than those who delayed treatment^9^. However, this study found that even with home prescriptions, 60% delayed treatment^9^. In a different study, patients with an action plan delayed treatment by an average of 6–7 days^10^. Other approaches to identifying COPD exacerbations such as telemonitoring programs have not consistently reduced ED visits or hospitalizations for COPD^11^. Therefore, a better understanding of why some patients delay treatment may help to improve COPD self-management programs and to help develop more effective telemonitoring programs.

The reasons for not reporting worsening symptoms to the healthcare team or delaying care may be that patients do not recognize symptoms of exacerbations^12^. Additionally, the desire to avoid hospitalizations may be a key factor in choosing not to seek care^13,14^. Qualitative studies of patients in the United Kingdom, Denmark, and the Netherlands found that barriers to seeking care included reluctance to see the doctor, not wanting to bother the doctor, lack of continuity of care, not knowing when it is appropriate to call, and difficulty traveling to the clinic^12,15,16^.

Given the potential benefits of early treatment during a COPD exacerbation, we utilized a qualitative descriptive design to understand how persons with COPD in a US health care system emotionally and cognitively perceived and managed their breathing symptoms, and the barriers and facilitators that led to seeking or delaying care for an acute exacerbation of COPD.

## Methods

Participants in this qualitative study were identified from a prospective observational study of 410 outpatients with COPD enrolled in primary care at two Veterans Affairs (VA) Medical Centers. Participants had COPD confirmed by spirometry (FEV_1_/FVC < 0.70), ≥1 treated COPD exacerbation in the previous year, and were English-speaking. Nursing-home residents and those with a diagnosis of dementia or Alzheimer’s disease were excluded. Institutional review board approval was obtained at VA Puget Sound and Eastern Colorado VA and all participants provided written informed consent; standard ethical and research-governance procedures were followed. We adhered to the consolidated criteria for reporting qualitative research (COREQ) guidelines (Supplement 1)^17^.

Participants were contacted every 2 weeks to screen for worsening COPD symptoms. Exacerbations were defined as new or worsening breathing symptoms that lasted more than 48 h^18,19^. Among the participants who experienced exacerbations during the study, we identified individuals eligible to participate in this qualitative study if they had exacerbations that met the following criteria: (i) untreated (did not seek care or take corticosteroids or antibiotics) exacerbations with worsening symptoms that persisted for ≥7 days and <6 weeks and were rated as moderately to extremely serious using the Response to Symptoms Questionnaire^20^ modified for COPD; (ii) treated with oral corticosteroids and/or antibiotics in the outpatient setting; (iii) treated in an urgent care or ED setting; or (iv) hospitalized. During January 2017 through February 2018, we purposively sampled from all 4 exacerbation categories soon after the exacerbation ended. Individuals were invited to participate in a phone-based, semistructured qualitative interview. Recruitment was discontinued when saturation occurred, and no new concepts or themes related to the study aims were identified^21^.

### Data collection

Interviews conducted by trained qualitative researchers (JY and CS) were digitally recorded and transcribed (interview length 32–65 min, average 48 min). The interview guide started with broad, open-ended questions, followed by more specific questions and probes to elicit thorough, detailed descriptions of exacerbation episodes. The questions examined perceived need for care for a COPD exacerbation based on Leventhal’s Common Sense Model to understand how individuals responded both cognitively and emotionally to worsening breathing symptoms^22^. Participants were asked to describe the course of their recent exacerbation including symptoms, their emotional and cognitive responses, coping strategies, symptom management, care-seeking, treatment received, involvement of others (such as caregivers, family, and friends), and comparison to past exacerbations and care-seeking behaviors (see Supplement 2 for Interview Guide). Interviewers also created written field notes for each interview to capture contextual information, encourage interviewer reflection, and facilitate interviewer technique improvement^23^.

### Analysis

Data were analyzed using inductive content analysis, an approach in which themes are derived from the data through open coding, code grouping, categorization, and identifying patterns of meaning across the data^24,25^. Experienced qualitative analysts (CS and JY) closely reviewed all transcripts and created an initial code list based on meaning units identified in the data. The coding framework was refined through discussion between the analysts and larger research team and coding discrepancies were resolved through consensus building. All data were coded using Atlas.ti qualitative software (version 8) and sorted into categories based on similarities and differences within and across interviews and themes were identified based on patterns in the data.

Interview data were iteratively revisited to refine themes, ensure findings were grounded in the data, and validate the results^26^. The entire study team, which included clinicians from a variety of disciplines and health-service researchers with expertise in COPD, reviewed analytic results at multiple time points to finalize themes, confirm the validity and credibility of findings, and identify representative quotes.

### Reporting summary

Further information on research design is available in the Nature Research Reporting Summary linked to this article.

## Results

Sixty participants were interviewed: (i) 15 untreated, (ii) 15 treated in the outpatient setting, (iii) 16 treated in the ED or urgent care clinic, and (iv) 14 hospitalized. The study cohort was mainly male (97%) with a mean age of 69.1 ± 6.9 years, mean FEV_1_ 1.42 (±0.63) liters, and mean mMRC dyspnea of 2.7 (±1.1) (Table 1).

**Table 1:** Demographics of interviewed participants.

	*N* = 60	
	N or mean	% or SD
*Demographics*		
Age	69.1	±6.9
Male gender	58	96.7
Race, Caucasian	47	78.3
Income >$20,000 per year	40	70.2
Currently employed	10	16.7
Education, college graduate	19	31.7
Live with others	43	71.7
Living as a couple	40	66.7
Dual Medicare and VA user	47	78.3
*COPD severity*		
FEV_1_ (liters)	1.42	±0.63
FEV_1_ % predicted	43.6	±19.4
FEV_1_ % predicted by severity category		
≥80% (Mild)	4	6.7
50-79% (Moderate)	16	26.7
30-49% (Severe)	25	41.7
0-29% (Very severe)	15	25.0
mMRC dyspnea scale	2.7	±1.1
*Type of exacerbation treatment during the one-year follow-up period*		
≥1 Untreated	47	78.3
≥1 treated as outpatient	30	50.0
≥1 urgent care or ED	23	38.3
≥1 Hospital	22	36.7
*COPD treatment*		
Chronic corticosteroid use, oral	7	11.7
Home oxygen therapy	31	51.7
Home nebulizer use	31	52.5
Pulmonary provider^a^	38	63.3
At-home prescription for corticosteroids and/or antibiotics	14	23.3
Prior participation in a pulmonary rehabilitation program	16	26.7
Enrolled in the VA care coordination home telehealth program for COPD	3	5.0
*Access to emergency care*		
Minutes to VA emergency department	62.7	38.7
Minutes to non-VA emergency department	15.9	9.4

^a^Either VA and/or non-VA pulmonary provider.

Four primary themes and 7 subthemes related to care seeking and delay of care were identified. The results are presented by theme and include select representative quotations (Table 2).

**Table 2:** Themes and corresponding exemplar quotes.

Quote #	Themes and quotes	Participant #	Type of exacerbation
**Theme 1: Access and attitudinal barriers contribute to reluctance to seek care**			
	***Attitudinal barriers to care***		
Q1	*I don’t like asking anybody for anything…they’d take me I know, but I just hate asking them. I don’t involve anybody in it. There’s family here, but I don’t involve them. It’s my problem*.	Pt 37	ED
Q2	*I don’t want to trouble the paramedics…or the hospital…every time I have these things, there’s a feeling of guilt…putting people through this …going to the doctor, going to the hospital, having to call 911, putting my wife through it*.	Pt 45	Untreated
	***Practical barriers to accessing in-person care***		
Q3	*It’s just a hassle - driving through traffic, the time it takes, paying for gas, missing work… I don’t go unless I absolutely have to*.	Pt 7	ED
Q4	*Yeah, just the inconvenience of going there*…*it’s not that I can’t go, there’s no one thing stopping me… I just don’t want to do it…it’s such a bother*.	Pt 11	ED
	***Heightened reluctance to seek care in ED settings, but few other options***		
Q5	*The ER, that’s the place to go only if you are in real dire straits, you don’t go there if your breathing is just bad, only if you absolutely can’t get a breath…so you put it off if you can*.	Pt 55	ED
Q6	*I don’t like going to the doctor but I hate going to the ER…I try to keep out of there as long as I possibly can*.	Pt 46	Untreated
Q7	*First I called them up [Primary Care office] but they said it would be a couple weeks before I could see anyone…couldn’t wait that long with my breathing*.	Pt 2	ED
Q8	*Sure, if I could call up and see someone [in primary care], I would do that but that’s not how it works…you can’t get in to see someone when you need it. They just say to go to the ED. … I don’t even call them anymore*.	Pt 20	Hospital
**Theme 2: Waiting is a typical response to new exacerbations**			
Q9	*I’ve been dealing with this so long…I always know when it’s getting started and something is coming on….always starts the same way…get tight in the chest, hard to take a deep breath*.	Pt 49	Untreated
	***“Trying everything first”***		
Q10	*I can tell when something is coming on so I do everything…my inhalers, my oxygen, my medications and I try and slow down and rest. Everything I can do myself first [before seeking care]*.	Pt 38	Outpatient treatment
Q11	*So I know what to do…I take my two inhalers, and if it still continues then I go up to the store and pick up some Claritin-D, some Sudafed, some Musinex…then after a while my breathing will open up and after a few days of those I’m a lot better. [LATER] Oh I’ve just figured out what works through trial and error…I’ve been dealing this this [COPD] long enough that I’ve just figured out what works for me*.	Pt 42	Untreated
Q12	*I have some medications my doctor gave me to use here when I need them [steroids and antibiotics]. I started on those after my breathing got bad and gunky…and I did all my other usual stuff like my inhalers. Then you wait and see what happens… You gotta give that stuff time to try and work it out*.	Pt 23	Hospital
Q13	*My doctor has given me things I can take if I need them [antibiotics] but I don’t like resorting to using them …don’t think it’s good to use them too much…don’t like how they make me feel and I don’t think it’s good for you*.	Pt 33	Outpatient treatment
	***“Waiting it Out”***		
Q14	*I just do all my usual stuff and wait for it to pass… sometimes it gets better and sometimes it don’t*.	Pt 3	ED
Q15	*Because you always seem to hope that it’s just a cough or cold, and then it just keeps getting bad and then you have to go to the doctor. You want to think it will get better, but usually it doesn’t*.	Pt 59	Outpatient treatment
	***Waiting but not waiting too long***		
Q16	*What happens is if I wait too long, if I fight it and try to do it with my own medications…then I have to go to the hospital…then I think I’ve waited too long. When I had to call the ambulance, I should’ve gone earlier*.	Pt 14	Hospital
Q17	*In my case, a lot of times I’d wait a week or two weeks before I’d go in. A lot of time I’d wait to the point where it’ll take forever to fix. If I hadn’t waited, if I’d gotten there sooner, it would’ve been fixed sooner and it wouldn’t have gotten so bad*.	Pt 18	Hospital
**Theme 3: Transitioning from waiting to care-seeking: The tipping point**			
Q18	*This time it didn’t get so bad. At about day seven, just when I was thinking I might need to head to the ED, I started breathing a little better. … Wasn’t nothing I did, just got lucky this time I guess*.	Pt 53	Untreated
Q19	*I always wait a week, that’s the time I give it each time my breathing gets bad. Then if it’s not better at a week I call the doctor*.	Pt 40	Untreated
Q20	*If what I’m doing just is not working and I’m using my inhalers here like crazy…and if it’s not working and I have nothing left to try, then I better get going*.	Pt 1	ED
Q21	*Yeah, when I couldn’t catch my breath even when I laid still…when I couldn’t even walk across the room or get to the bathroom, I decided this is something I couldn’t handle by myself. So I went to the ER*.	Pt 10	ED
Q22	*I can usually tell by what I’m coughing up, like a rainbow. And if it gets to be a certain color, I call my doctors*.	Pt 28	Outpatient treatment
Q23	*I turned my machine all the way up and still couldn’t breathe…my rescue inhaler didn’t work. I was gasping for air and got all panicky which made it worse, so I ended up taking an ambulance to the hospital*.	Pt 19	Hospital
Q24	*I always give it about a week to get better on its own. But then I got tired of not being able to breathe and thought I’d better do something about this*.	Pt 38	Outpatient treatment
Q25	*Well honestly, I only called the doctor because she [wife] was on my case so bad to get seen. So I called and ended up going in to Urgent Care that night*.	Pt 12	ED
Q26	*My wife was really fed up with my bad breathing…I didn’t want to go to the ER but she said ‘that’s enough of that’ and called 911*.	Pt 4	ED
**Theme 4: Learning from and avoiding worse outcomes**			
Q27	*Well I’ve played that waiting game before and ended up in the hospital….this time I saw it coming on and went on in to get checked out*.	Pt 9	ED
Q28	*I didn’t hesitate [to seek care] this time. I don’t mess around with my symptoms and hope they will go away like I used to. Now I want to nip it in the bud, go in there earlier and take care of things so I don’t end up so sick…don’t need another case of pneumonia*.	Pt 34	Outpatient treatment
	***Clinician role in encouraging care seeking***		
Q29	*My doctor [pulmonologist] got on me about it…told me not to wait so long. Don’t try to get in and see her, just go into the ER if I’m having any problems*.	Pt 39	Outpatient treatment
Q30	*[PCP] told me I was doing damage to myself by not getting in there sooner and I might be hurting my lungs or doing other bad stuff to my body. He said as soon as my breathing gets bad I’d better get myself to the ER…now I try and make myself get in there sooner*.	Pt 2	ED
Q31	*I always call to see if I can get into my PCP and I’ll wait to see her if I can… sometimes can’t get in and then I end up waiting, you know, and then end up at the ER anyway…if only I could get in to see her then I wouldn’t do all that waiting and wouldn’t have to go there [ER]…that’s what I want to happen*.	Pt 54	ED

### Theme 1: Attitudinal and access barriers contributed to reluctance to seek care

When recounting exacerbation episodes, participants consistently reported a reluctance to seek care and the desire to “put off” care-seeking. This reluctance was based on a combination of attitudinal and access-related barriers to care.

#### Attitudinal barriers to care

Participants described avoiding care-seeking because of an attitude of self-reliance and not wanting to “bother” or “burden” others. Wanting to “go it alone” and concerns about the impact of exacerbations on family and clinicians were commonly described as contributing to hesitancy to seek care (*Q1, Q2*).

#### Practical barriers to accessing in-person care

The “hassle” or “inconvenience” of seeking care was commonly cited as a motivation for delaying care-seeking and comprised a combination of practical issues such as distance to care, concerns with driving during an exacerbation, time associated with seeking and receiving care, and missing work or neglecting other responsibilities. Although each of these factors alone was not a significant barrier to care, participants described their additive nature as contributing to a pervasive reluctance toward care-seeking (*Q3, Q4*).

#### Heightened reluctance to seek care in ED settings, but few other options

Many participants described reluctance to seek care in emergency settings due to amplified practical concerns (such as greater travel distance and time spent waiting for treatment) and a belief that the ED is appropriate only in “true emergencies” like severe shortness of breath (*Q5*). For some, this reluctance impacted care-seeking and translated into increased delays in care (*Q6*). Although individuals frequently reported wanting to receive treatment in outpatient settings, many said they encountered wait times for outpatient appointments that precluded being seen in primary care during an acute COPD exacerbation (*Q7*). Additionally, many participants reported calling for a primary care appointment only to be instructed to seek care in the ED instead. Some participants explained that a history of being referred from primary to emergency care made it less likely that they would contact primary care for future exacerbations (*Q8*).

### Theme 2: Waiting is a typical response to new exacerbations

Participants described being aware when a worsening breathing episode began and recognized early exacerbation symptoms such as fatigue, shortness of breath, increased difficulty in breathing, chest tightness, increased sputum production, and/or coughing. Many participants described having their own typical symptom-presentation patterns, and attributed “knowing when something [exacerbation] is coming on” to their history of living with COPD and their experience with recurrent exacerbations (*Q9*). Almost all participants described waiting as a normative, typical response to the onset of a new exacerbation and new symptoms, with the following subthemes:

#### “Trying everything first”

All participants described addressing new exacerbation symptoms with home-based treatments or symptom management prior to care-seeking. Patients often described developing their home-treatment approach through experimentation and experience; few reported having been provided with or following specific clinical instruction, although a minority of patients had been prescribed at-home medications (prednisone or antibiotics) to take for exacerbations. This “first line of defense” included using over-the-counter and on-hand prescribed medications, increasing home oxygen levels, increasing inhaler use, and/or adding “stronger” respiratory treatments like nebulizers and resting (*Q10*) (*Q11*). Participants described implementing these treatments singly or in combination, and waiting to see what, if any, effect they had on symptoms. For some, a variety of management approaches were tried, or layered together, over time (*Q12*). The individuals who were prescribed prednisone or antibiotics to have on-hand often reported trying other self-management approaches before “resorting” to taking them during an exacerbation due to concerns about overreliance on antibiotics or steroids and perceived negative side effects (*Q13*).

#### “Waiting it Out”

Participants also commonly described waiting to see if their symptoms would subside in the hope of avoiding care-seeking. Some explained that the courses of their exacerbations were unpredictable and varied, with some resolving without incident and others worsening and requiring medical attention (*Q14*). However, many participants acknowledged that hopes of avoiding care-seeking and the desire to “wait it out” often led them to overestimate the chances that episodes would improve and to ignore or downplay worsening symptoms (*Q15*).

#### Waiting but not waiting too long

Many participants acknowledged potential negative outcomes related to delayed care-seeking and described a balance between wanting to wait for care but not wanting to “wait too long.” “Waiting too long” was defined as allowing episodes to progress to the point of negative outcomes such as urgent care-seeking (e.g., panicking, activating emergency medical services [calling 911], and having to be rushed to the ED), advanced illness (e.g., pneumonia, passing out from breathlessness), and/or hospitalization (*Q16*). Many described a desire to wait until the symptoms were “bad enough” to necessitate medical treatment but not serious enough to end in crisis. However, waiting too long was common and many described episodes in which waiting resulted in negative outcomes (*Q17*).

### Theme 3: Transitioning from waiting to care-seeking: the tipping point

All participants who sought treatment described a tipping point at which they transitioned from waiting and managing exacerbation symptoms to active care-seeking. Participants who did not seek care reported that their symptoms improved and never reached a tipping point, although some acknowledged that an episode could have easily gotten worse and considered themselves “lucky” that they improved (*Q18*). Each person who sought care had their own criteria for care-seeking, and participants often reported applying their individual tipping point consistently across exacerbations (*Q19*). Individuals initiated care-seeking in the following four common circumstances:

#### Self-management strategies were exhausted

Some participants reported seeking care when symptoms worsened or failed to improve, despite best efforts at home-based treatment of their exacerbation episode. Individuals often described seeking care only when they had used all their available inhalers and medications and had “nothing left to try” (*Q20*).

#### Severe or worrisome symptoms

Participants commonly described waiting until their symptoms worsened and became urgent, unbearable, or “bad enough” to necessitate care-seeking. Extreme breathlessness (e.g., gasping for breath, unable to walk across the room) (*Q21*), and unusual, observable, “worrisome signs” (e.g., sputum color, temperature, and coughing leading to bleeding or vomiting) (*Q22*), were commonly described as triggering care-seeking. Breathlessness was sometimes accompanied by panic or anxiety, which often resulted in urgent care-seeking, such as calling 911 (*Q23*).

#### Episodes went on longer than anticipated

Participants often described a typical time they waited before seeking care, and episodes that exceeded this duration prompted care-seeking. While most reported they would wait approximately a week before seeking care, the duration of symptoms prior to care-seeking ranged widely from 3 days to 2½ weeks (*Q24*).

#### A family member encouraged or insisted upon care-seeking

Some participants described seeking care only with the encouragement or insistence of family members (e.g., wives or adult children). Often, participants did not believe their symptoms warranted medical treatment and acknowledged that they sought care only to appease or address the concerns of others (*Q25*). Although family-member involvement typically prompted participant care-seeking behaviors, a few participants described episodes in which family members sought care (such as calling a doctor or 911) without the participant’s instruction (*Q26*).

### Theme 4: Learning from and avoiding worse outcomes

Although many participants described consistently delaying care-seeking during COPD exacerbations, some reported that their care-seeking patterns evolved over time. The desire to avoid repeating past experiences with worse outcomes like hospitalization and calling 911 sometimes served as a powerful motivator toward earlier care-seeking (*Q27*). Some explained that they learned to be more proactive in their care-seeking in order to “nip it [exacerbation] in the bud” and avoid more severe illness like pneumonia (*Q28*).

#### Clinician role in encouraging care-seeking

A few participants reported that clinicians played an important role in underscoring the impact of worse outcomes and encouraging earlier care-seeking. Clinical encounters following a negative outcome like hospitalization or calling 911 affected some individuals’ understanding of when, where, and why to seek earlier care during an exacerbation (*Q29, Q30*). Some participants stated that having access to a primary care provider visit within a few days of calling, as opposed to needing to go to the ED, would facilitate earlier treatment (*Q31*).

## Discussion

In this study, a reluctance to seek care was a typical or normative response of many persons with COPD when they developed symptoms of an exacerbation. Participants generally sought care when self-management strategies were exhausted, severe symptoms developed, episode duration was longer than expected, and/or family members encouraged care-seeking. Participants did not seek care until they reached a tipping point when their perceived need for care overcame the attitudinal and access barriers they faced.

Similar to our results, a reluctance to seek care has been documented in several qualitative studies of individuals with COPD in which themes of stoicism and wishing to not bother their providers contributes to delays in seeking care^12,15,16,27^. Our study also suggests that treatment setting can impact an individual’s reluctance or willingness to seek care. Many participants described a heightened reluctance to seek care in the ED for practical and attitudinal reasons and preferred to be seen in a primary care setting during an exacerbation. However, participants often were unable to make timely primary care appointments and were told to seek care in the ED, which they commonly sought to avoid.

Prior studies found that distinguishing between COPD exacerbations and daily fluctuation of baseline symptoms may be challenging, especially for older adults^28^. Qualitative studies of individuals with heart failure^29^ and COPD^30^ found that individuals who had difficulty recognizing symptoms and/or attributing them to an exacerbation often delayed seeking care. However, a study of COPD patients found that patients were generally able to identify exacerbations based on worsening symptoms^31^. Similarly, in our study, participants did not have difficulty recognizing exacerbation symptoms, and were able to readily distinguish the typical symptom onset from their baseline breathing. All participants in the study cohort experienced at least one treated exacerbation in the year prior to the study, which may have made it more likely to recognize new exacerbations.

Upon recognizing the symptoms, participants described a typical approach of waiting to see whether exacerbation symptoms would respond to at-home treatments that were often based on trial-and-error approaches through experience with prior exacerbations rather than clinical instruction. Participants in our study were not enrolled in a formal COPD self-management program, although 27% had previously participated in a pulmonary rehabilitation program, 5% were enrolled in a VA telemonitoring program, and 23% reported receiving a home prescription for prednisone or antibiotics. Waiting, often for one or more weeks, until their symptoms either improved or became unbearable or severe was part of their self-management strategy, and at times led to a crisis requiring individuals to be hospitalized. Williams and colleagues reported that individuals’ prior experience managing exacerbations played an important role in determining how they identified and managed exacerbations. Similar to our study, participants preferred to manage their symptoms by themselves at home and felt confident monitoring their symptoms based on their experiential knowledge of living with COPD for many years^30^^,^^31^.

Laue and colleagues reported that COPD exacerbation self-treatment was a means of avoiding seeking medical care. When at-home treatment failed, some patients further delayed seeking care as they were worried it would be viewed as a personal failure^32^. A study by Korpershoek and colleagues found that some patients recognized exacerbation symptoms early and were likely to initiate prompt self-management and follow-up with a healthcare provider, whereas others lacked self-management strategies and were less likely to seek care^30^. The factors associated with care-seeking included self-empowerment and trusting their primary care provider whereas those who delayed care reported being stubborn or not wanting to bother their provider, as well as ambivalence toward treatment due to negative side effects of prednisone^30^.

Although earlier treatment of COPD exacerbations can lead to more rapid resolution of worsened breathing symptoms and may reduce the likelihood of ED visits and hospitalizations^1,9^, even with self-management training, some patients still delay care^9,10^. Therefore, it is important to incorporate individuals’ experiences with prior exacerbations to reduce barriers to seeking care sooner. Our study suggests that strategies to address attitudinal barriers such as self-reliance and wanting to avoid being a burden to others may be needed. For example, promising results from an open trial of motivational interviewing (MI)-based health coaching^33^ and a small trial of MI to improve treatment adherence suggest that MI may help address these attitudinal barriers^34^.

In addition to addressing patient characteristics, our findings suggest that healthcare systems need to address the “hassle” aspects of seeking care and other perceived logistical barriers to accessing care to make it easier for individuals to choose to seek care earlier. Such system-level improvements might include increasing options for evaluation and treatment in outpatient clinical settings, including options for virtual care, to provide alternatives to going to the ED. Additionally, follow-up visits after hospitalizations and ED visits may provide opportunities to help individuals with COPD improve self-management skills and care-seeking behaviors.

Another approach to encourage early care-seeking is to incorporate family caregivers who are important in COPD management and treatment planning^35,36^. Providing education on when and where to seek treatment, and support for family members in their caregiving roles may be beneficial^37^. Given that most participants in our study were male veterans, and many family caregivers are likely female, care-seeking strategies may need to take these gender dynamics into account. However, further exploration of the efficacy and benefits of this strategy is needed as caregivers may lack agency, knowledge, and support to best help individuals with COPD, and attempts to influence earlier care-seeking could cause relational tension or conflict^38^.

It is possible that underlying attitudes around care-seeking during an exacerbation may differ among people who regularly seek care compared with those who opt to “wait-it-out” without taking corticosteroids or antibiotics or seeing a provider. A key strength of this study is that we interviewed participants who received different types of care for their exacerbations, including those who successfully recovered without formal treatment, those treated as outpatients, and those with more severe exacerbations treated in the ED or as inpatients. We noted themes shared among the four treatment groups around the desire to wait and attempt to manage symptoms at home. Participants who had not sought care for an exacerbation stated that they had not reached the tipping point at which they would seek care.

A limitation of this study was that most participants were male veterans, and these findings may not generalize to the greater population of individuals with COPD. Older male veterans represent a cultural group that includes traits associated with masculinity and military culture such as stoicism, concealing vulnerability and non-help-seeking behaviors^39,40^. While similar traits of stoicism and self-reliance have been noted among nonveterans with COPD^27,41^, differences in care-seeking attitudes may exist. The risk of exacerbations and care-seeking behaviors of female patients with COPD may be different as well^42,43^. Indeed, one study found that women with COPD were more likely to delay presenting to the ED compared with men^44^, while another study of COPD hospitalizations at VA facilities found that compared with men, women were less likely to receive some inhaled medications, with shorter hospital stays and lower readmission rates^45^. It remains to be seen, however, whether the diverse population of people who live with COPD would report the same kinds of attitudes about care-seeking as we found in our US sample of predominantly male veterans. As COPD is a global public health challenge, further research is needed to understand how contextual factors such as different social conditions or cultures may influence seeking or delaying care and will be vital in shaping intervention strategies that are applicable to the diverse groups of people with COPD.

In summary, we found that reluctance to seeking care is a common response to exacerbations. Self-management approaches based on prior exacerbation experiences were often used before seeking care, and failure of these strategies was pivotal in the decision of when and where to seek care. Attitudinal barriers such as self-reliance along with perceived barriers to access to care, including the desire to avoid the emergency room, were important factors in delaying care. Interventions that seek to understand patients’ lived experience with exacerbations, their attitudes toward seeking care, and address potentially suboptimal self-management approaches combined with healthcare-system changes to improve access to nonemergent outpatient treatment, may help to facilitate earlier intervention for COPD exacerbations.

## Supplementary information

Supplementary Information

REPORTING SUMMARY

## Data Availability

Due to ethical restrictions, we are unable to share data publicly because the data contain potentially identifying and/or sensitive patient information.
