# Author Correction: Care-seeking and delay of care during COPD exacerbations

**DOI:** 10.1038/s41533-022-00279-7

**Published:** 2022-04-24

**Authors:** Emily R. Locke, Jessica P. Young, Catherine Battaglia, Tracy L. Simpson, Ranak Trivedi, Carol Simons, John C. Fortney, Paul Hebert, Erik R. Swenson, Jeffrey Edelman, Vincent S. Fan

**Affiliations:** 1 Center of Innovation for Veteran-Centered and Value-Driven Care VA Puget Sound Health Care System, Seattle, WA United States; 2 Eastern Colorado VA Health Care System, Aurora, CO United States; 3 University of Colorado Anschutz Medical Campus, Aurora, CO United States; 4 Center of Excellence in Substance Addiction Treatment and Education (CESATE), VA Puget Sound Health Care System, Seattle, WA United States; 5 Department of Psychiatry, University of Washington School of Medicine, Seattle, WA United States; 6 Center for Innovation to Implementation, VA Palo Alto Health Care System, Palo Alto, CA United States; 7 Department of Psychiatry and Behavioral Sciences, Stanford University, Stanford, CA United States; 8 Department of Health Systems and Population Health, University of Washington, Seattle, WA United States; 9 Pulmonary and Critical Care, VA Puget Sound Health Care System, Seattle, WA United States; 10 Department of Medicine, University of Washington, Seattle, WA United States

**Keywords:** Chronic obstructive pulmonary disease, Respiratory signs and symptoms, Outcomes research, Health services

Correction to: *npj Primary Care Respiratory Medicine* 10.1038/s41533-022-00269-9, published online 15 February 2022

In this Article, a disclaimer was missing from the Acknowledgements section that should have stated: “The views expressed in this article are those of the authors and do not necessarily reflect the position or policy of the Department of Veterans Affairs or the United States Government.” This has now been updated.

