# Supplementary material for: Care-seeking and delay of care during COPD exacerbations
Source: NPJ Prim Care Respir Med. 2022 Feb 15;32:7. doi: 10.1038/s41533-022-00269-9 (PMC8847354; doi:10.1038/s41533-022-00269-9)
Supplement: Supplementary file 2 — REPORTING SUMMARY [file 41533_2022_269_MOESM2_ESM.pdf]

nature portfolio

Corresponding author(s): Emily Locke

Last updated by author(s): Nov 9, 2021

Reporting Summary

Nature Portfolio wishes to improve the reproducibility of the work that we publish. This form provides structure for consistency and transparency in reporting. For further information on Nature Portfolio policies, see our Editorial Policies and the Editorial Policy Checklist.

Statistics

For all statistical analyses, confirm that the following items are present in the figure legend, table legend, main text, or Methods section.

n/a	Confirmed	
☒	☐	The exact sample size (n) for each experimental group/condition, given as a discrete number and unit of measurement
☒	☐	A statement on whether measurements were taken from distinct samples or whether the same sample was measured repeatedly
☒	☐	The statistical test(s) used AND whether they are one- or two-sided<br>*Only common tests should be described solely by name; describe more complex techniques in the Methods section.*
☒	☐	A description of all covariates tested
☒	☐	A description of any assumptions or corrections, such as tests of normality and adjustment for multiple comparisons
☒	☐	A full description of the statistical parameters including central tendency (e.g. means) or other basic estimates (e.g. regression coefficient) AND variation (e.g. standard deviation) or associated estimates of uncertainty (e.g. confidence intervals)
☒	☐	For null hypothesis testing, the test statistic (e.g. F, t, r) with confidence intervals, effect sizes, degrees of freedom and P value noted<br>*Give P values as exact values whenever suitable.*
☒	☐	For Bayesian analysis, information on the choice of priors and Markov chain Monte Carlo settings
☒	☐	For hierarchical and complex designs, identification of the appropriate level for tests and full reporting of outcomes
☒	☐	Estimates of effect sizes (e.g. Cohen's d, Pearson's r), indicating how they were calculated

Our web collection on statistics for biologists contains articles on many of the points above.

Software and code

Policy information about availability of computer code

Data collection	*Provide a description of all commercial, open source and custom code used to collect the data in this study, specifying the version used OR state that no software was used.*
Data analysis	*Provide a description of all commercial, open source and custom code used to analyse the data in this study, specifying the version used OR state that no software was used.*

For manuscripts utilizing custom algorithms or software that are central to the research but not yet described in published literature, software must be made available to editors and reviewers. We strongly encourage code deposition in a community repository (e.g. GitHub). See the Nature Portfolio guidelines for submitting code & software for further information.

Data

Policy information about availability of data

All manuscripts must include a data availability statement. This statement should provide the following information, where applicable:

- Accession codes, unique identifiers, or web links for publicly available datasets
- A description of any restrictions on data availability
- For clinical datasets or third party data, please ensure that the statement adheres to our policy

Due to ethical restrictions we are unable to share data publicly because the data contains potentially identifying and/or sensitive patient information.

Field-specific reporting

Please select the one below that is the best fit for your research. If you are not sure, read the appropriate sections before making your selection.

☐ Life sciences ☒ Behavioural & social sciences ☐ Ecological, evolutionary & environmental sciences

For a reference copy of the document with all sections, see nature.com/documents/nr-reporting-summary-flat.pdf

Behavioural & social sciences study design

All studies must disclose on these points even when the disclosure is negative.

Study description	Qualitative study of semi-structured interviews
Research sample	Individuals diagnosed with COPD enrolled in primary care at Veterans Affairs (VA) Puget Sound and VA Eastern Colorado Medical Centers. Participants had COPD confirmed by spirometry (FEV1/FVC < 0.70), ≥ 1 treated COPD exacerbation in the previous year, and were English-speaking. Nursing home residents and those with a diagnosis of dementia or Alzheimer's disease were excluded.
Sampling strategy	Participants in this study were identified from a prospective observational study of 410 COPD patients. Participants were contacted every 2 weeks to screen for exacerbations. We purposively sampled from 4 exacerbation categories: 1) untreated, 2) treated with prednisone and/or antibiotics as an outpatient, 3) treated in the urgent care/emergency department setting, or 4) hospitalized. Individuals were invited to participate in a phone-based, semi-structured qualitative interview. Recruitment was discontinued when saturation occurred, and no new concepts or themes related to the study aims were identified
Data collection	Phone interviews conducted by trained qualitative researchers (JY and CS) were digitally recorded and transcribed. To our knowledge there were no third persons influencing the study participants; however, given that interviews took place over the phone, we cannot be absolutely certain that third parties were not present. Interviewers created written field notes for each interview to capture contextual information and encourage interview reflection.
Timing	Interviews were conducted January 2017 through February 2018.
Data exclusions	No data were excluded.
Non-participation	Participation in the qualitative interview was not required as part of one's participation in the parent study. Unfortunately we did not track how many participants who were invited to participate in a qualitative interview declined to participate.
Randomization	This was an observational study and there was no randomization.

Reporting for specific materials, systems and methods

We require information from authors about some types of materials, experimental systems and methods used in many studies. Here, indicate whether each material, system or method listed is relevant to your study. If you are not sure if a list item applies to your research, read the appropriate section before selecting a response.

Materials & experimental systems

n/a	Involved in the study
☒	☐ Antibodies
☒	☐ Eukaryotic cell lines
☒	☐ Palaeontology and archaeology
☒	☐ Animals and other organisms
☐	☒ Human research participants
☐	☒ Clinical data
☒	☐ Dual use research of concern

Methods

n/a	Involved in the study
☒	☐ ChIP-seq
☒	☐ Flow cytometry
☒	☐ MRI-based neuroimaging

Human research participants

Policy information about studies involving human research participants

Population characteristics	Participants in the parent study, "Understanding Patient Management of COPD Exacerbations", had COPD confirmed by spirometry (FEV1/FVC < 0.70), ≥ 1 treated COPD exacerbation in the previous year, and were English-speaking. Nursing home residents and those with a diagnosis of dementia or Alzheimer's disease were excluded. The study cohort was mainly male (97%) with a mean age of 69.1 ± 6.9 years, mean FEV1 1.42 (±0.63) liters and mean mMRC dyspnea of 2.7 (±1.1).
Recruitment	Participants in the parent study, "Understanding Patient Management of COPD Exacerbations", who experienced a COPD exacerbation were invited to participate in the qualitative study if they had exacerbations in one of the following four

	categories: i) untreated exacerbations (N=15), ii) exacerbations treated in the outpatient setting (N=15), iii) treated in the emergency department or urgent care clinic (N=16), and iv) hospitalized (N=14).
Ethics oversight	Institutional Review Board approval was obtained at VA Puget Sound and Eastern Colorado VA.

Note that full information on the approval of the study protocol must also be provided in the manuscript.

Clinical data

Policy information about clinical studies

All manuscripts should comply with the ICMJE guidelines for publication of clinical research and a completed CONSORT checklist must be included with all submissions.

Clinical trial registration	*Provide the trial registration number from ClinicalTrials.gov or an equivalent agency.*
Study protocol	*Note where the full trial protocol can be accessed OR if not available, explain why.*
Data collection	*Describe the settings and locales of data collection, noting the time periods of recruitment and data collection.*
Outcomes	*Describe how you pre-defined primary and secondary outcome measures and how you assessed these measures.*
